# Response of the mosquito protein interaction network to dengue infection

**DOI:** 10.1186/1471-2164-11-380

**Published:** 2010-06-16

**Authors:** Xiang Guo, Yao Xu, Guowu Bian, Andrew D Pike, Yan Xie, Zhiyong Xi

**Affiliations:** 1 Department of Entomology and Genetics Program, Michigan State University, East Lansing, Michigan 48824, USA; 2 J Craig Venter Institute, Rockville, MD 20850, USA; 3 Center for Statistical Training & Consulting, Michigan State University, East Lansing, Michigan 48824, USA; 4 Advanced Biomedical Computing Center, SAIC-Frederick Inc, NCI-Frederick, MD 21702, USA

## Abstract

**Background:**

Two fifths of the world's population is at risk from dengue. The absence of effective drugs and vaccines leaves vector control as the primary intervention tool. Understanding dengue virus (DENV) host interactions is essential for the development of novel control strategies. The availability of genome sequences for both human and mosquito host greatly facilitates genome-wide studies of DENV-host interactions.

**Results:**

We developed the first draft of the mosquito protein interaction network using a computational approach. The weighted network includes 4,214 *Aedes aegypti *proteins with 10,209 interactions, among which 3,500 proteins are connected into an interconnected scale-free network. We demonstrated the application of this network for the further annotation of mosquito proteins and dissection of pathway crosstalk. Using three datasets based on physical interaction assays, genome-wide RNA interference (RNAi) screens and microarray assays, we identified 714 putative DENV-associated mosquito proteins. An integrated analysis of these proteins in the network highlighted four regions consisting of highly interconnected proteins with closely related functions in each of replication/transcription/translation (RTT), immunity, transport and metabolism. Putative DENV-associated proteins were further selected for validation by RNAi-mediated gene silencing, and dengue viral titer in mosquito midguts was significantly reduced for five out of ten (50.0%) randomly selected genes.

**Conclusions:**

Our results indicate the presence of common host requirements for DENV in mosquitoes and humans. We discuss the significance of our findings for pharmacological intervention and genetic modification of mosquitoes for blocking dengue transmission.

## Background

Dengue fever and associated dengue hemorrhagic fever are emerging globally as the most important arboviral disease threatening human populations. Dengue virus (DENV) is transmitted to humans by the mosquitoes *Aedes aegypti *and *Aedes albopictus*. When ingested into the mosquito midgut with blood, DENV first interacts with midgut cell membrane receptors and then enters the cells through receptor-mediated endocytosis. Following replication in midguts, DENV disseminates to salivary glands for transmission to human. In the mosquito, DENV is attacked by the mosquito innate immune system, including RNAi and Toll pathway [1,2]. In addition, the effects of several host genes were reported to determine the mosquito's susceptibility to dengue infection [3].

DENV belongs to the genus *Flavivirus *of the *Flaviviridae *family. Flaviviruses are small, enveloped viruses with a single-stranded positive-sense RNA, which can be translated into a single polyprotein by host cell proteins. The polyprotein is then cleaved into individual proteins by both viral and host proteases [4]. Three structural proteins (capsid, pre-membrane, and envelope) constitute the virus particle. Seven non-structural proteins (NS1, NS2A, NS2B, NS3, NS4A, NS4B, NS5), 5' and 3' untranslated regions (UTR) are involved in RNA replication. The N-terminus of NS3 codes a serine protease essential for virus replication, and the central domain of NS2B is a cofactor for the NS3 serine protease. Virus-host interactions start with binding of the envelope protein E to a cellular receptor. Then, a complex interplay of viral and cellular proteins modulates the process of viral translation, replication, and assembly [5]. Most previous studies on the replication mechanisms of DENV have been conducted in human cells. These studies identified putative cellular receptors and other host proteins that may interact with viral proteins or RNAs [6]. In contrast, relatively little has been done to elucidate how DENV utilizes the cellular factors to enter and replicate in mosquito cells. It remains unclear which virus replication features are responsible for efficient virus passage between these different host systems.

Availability of genome sequences for both humans and the mosquito vector, *Ae. aegypti*, now provides us novel opportunities to identify the host factors that are required for dengue infection. Recently, microarray and RNAi screens were used for genome-wide studies of interactions between DENV and their hosts. They led to identification of a number of cellular factors that can determine the susceptibility and resistance of both hosts to DENV [2,7]. These results also emphasize the complexity of interactions between flaviviruses and its hosts. Understanding of such interactions requires systematic and comprehensive studies of the host biology as it relates to viral infections. Analysis of protein interaction network is one way to dissect such complex interactions and has been broadly used to study the systems biology of host-pathogen interactions [8-11].

This systems biology approach requires the construction of signaling and regulatory networks. Physical interactions between two proteins are the basis for protein complexes and signaling networks, while transcription factors regulate gene transcription by binding to their target DNA sequences in the context of intricate transcriptional regulatory networks. Large amounts of high-throughput data are now available in several model organisms for protein-protein interactions [12-14], which have been extensively used in network reconstruction and systems biology studies. However, the high cost of experimental methods prevents large scale interaction network mapping of newly sequenced genomes. Computational approaches provide a viable alternative for protein-protein interaction mapping. Experimental interactomes in model organisms can be transferred to other species based on homology information. This interolog approach has been widely used to build protein interaction networks in humans [15,16] and other species [17]. Although unique interactions in a new species can not be found by the interolog method, the predicted interactomes may reveal conserved complexes and pathways, and serve as important tools for annotating uncharacterized proteins [18]. The same concept can be applied to predict interactions between proteins in different species.

Uetz et al. predicted interactions between herpesvirus and human proteins based on known interacting orthologs in model organisms, and co-immuniprecipitation confirmed 68% of their predictions [19]. DENV consists of only 10 proteins, which lack orthologs in model organisms. However, viruses in the genus *Flavivirus *often target the same host proteins, so known flavivirus-host interactions may be used to predict DENV-mosquito protein interactions. Recently, a high throughput RNAi screen in human cells identified 283 host susceptibility factors and 22 host resistant factors for the infection of West Nile virus (WNV; genus *Flavivirus*, family *Flaviviridae*). More importantly, targeted silencing of the WNV host factors found that half of the 283 susceptibility factors and all of the 22 resistant factors affected dengue infection in the same manner as they affected WNV infection [20]. Additional independent studies also provide evidence that multiple flaviviruses, including WNV, Japanese encephalitis (JEV) and DENV, target the same host proteins [21,22]. It was reported that these flaviviruses share a similar genomic organization and replication strategy although they cause a range of distinct clinical diseases in humans [6].

Evidence also indicates the notable conservation of required dengue host factors between insects and humans. Recently, a genome-wide RNA interference screen in *Drosophila melanogaster *cells identified 116 dengue virus host factors. Among them, 82 have human homologues. Using targeted siRNA, 51.2% of them proved to be dengue host factors in humans [7]. A number of conserved host factors were also identified in several earlier studies. In both mosquitoes and humans, La protein interacts with 5' and 3' ends of dengue viral RNA and two nonstructural proteins NS3 and NS5. Such interactions seem important for assembly of a functional replication complex of dengue virus [23,24]. Laminin-binding protein and heat shock proteins were also indicated to mediate the dengue infection in both humans and mosquitoes [21,25-28]. Moreover, a similar internalization route (clathrin-mediated endocytosis) was used by dengue viruses to enter into human and mosquito cells.

In this study, we have developed the mosquito protein interaction network based on large-scale protein interaction datasets in *Saccharomyces cerevisiae, Caenorhabditis elegans *and *Drosophila melanogaster*. 714 flavivirus-associated mosquito proteins were inferred from three data sources based on physical interaction assays, genome-wide RNAi screens and microarray results. An integrated analysis of dengue-associated protein interaction sub-networks highlighted four regions consisting of highly interconnected proteins with closely related functions. Finally, we verified that five out of ten (50.0%) randomly selected genes were required for DENV infection in the main mosquito vector, *Ae. aegypti*, by RNAi-mediated gene silencing.

## Results and Discussion

### Construction of the first-draft mosquito protein interaction map

Reliable protein interaction datasets in model organisms are the basis of the interolog method. Because large-scale protein interaction mapping generates a high number of false positive interactions, it is essential to assess the quality of experimentally determined protein interactions. To generate the draft mosquito interaction network, we selected different interaction datasets with available confidence scores or rankings. The dataset in *S. cerevisiae *includes 47,783 interactions identified by co-immunoprecipitation and yeast two-hybrid screens. Each interaction is associated with a confidence score estimated by the logistic regression model [29]. The two-hybrid-based dataset in *D. melanogaster *includes 20,405 interactions with a confidence score estimated by a generalized linear model [12]. The dataset in *C. elegans *(4,736 interactions) includes literature-mined data and two-hybrid generated data divided into three confidence classes [13]. For all three datasets, confidence metrics were generated using only statistical and topological predictors, such as the number of interaction partners and a local clustering coefficient. These scores were combined with the InParanoid [30] score to generate confidence values for predicted interactions in *Ae. aegypti*.

**Figure 1:**
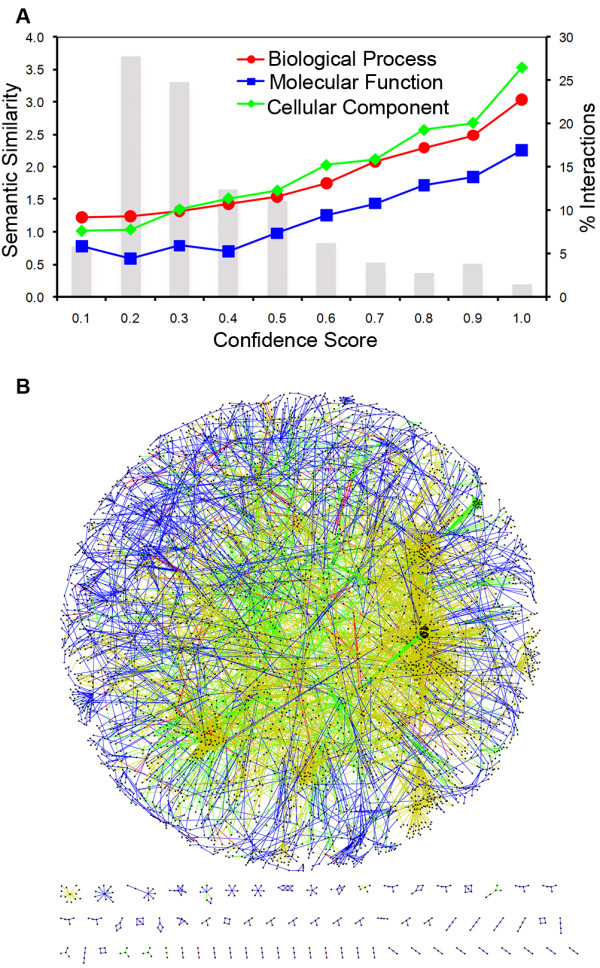
**Construction of the mosquito protein interaction network**. (A). Semantic similarity and interaction confidence of putative interacting proteins in *Ae. aegypti*. Protein interactions were binned based on their confidence scores. The average semantic similarity values in each bin were shown for all three GO sub-categories: biological process, molecular function, and cellular component. Percentage of interactions in each bin was shown as a histogram bar. (B) Visualization of the predicted mosquito protein interaction network. Colors are used to show the origin of the inferred interactions. *D. melanogaster*: blue, *C. elegans: *green, *S. cerevisiae: *yellow, multiple sources: red.

Our predictions resulted in 64,449 interactions for 6,263 mosquito proteins, for which the average confidence score is 0.32 (Additional file 1). To test the effectiveness of our confidence scoring scheme, we examined the association of semantic similarity and confidence score for the predicted interactions. Interacting proteins often participate in the same molecular pathway and are located in the same cellular compartment. Functional relatedness has been shown to be the best feature in discriminating true interactions from noise [31], and our previous work demonstrated the advantage of Gene Ontology (GO)-based similarity measures in protein interaction validation [32]. Among our predicted interactions, there are 32,826, 36,132, and 20,127 interacting protein pairs with annotation of three GO sub-categories: biological process, molecular function, and cellular component, respectively. Our results indicate that high confidence interactions correlate with protein pairs with high semantic similarity, suggesting the effectiveness of our confidence scoring scheme and the quality of our predictions (Fig. 1A). Because a large number of GO annotations for *Ae. agypti *proteins are based on sequence homology to *D. melanogaster*, the high correlation between semantic similarity and confidence score of interacting proteins predicted from *Drosophila *may simply reflect their correlation in *Drosophila*. Thus, we have repeated the analysis using interactions predicted from yeast alone. This dataset also shows an apparent correlation between interaction confidence and functional similarity (Additional file 2), indicating the effectiveness of our confidence scoring scheme.

Using predicted interactions with confidence values more than 0.5, we constructed a weighted protein interaction network for *Ae. aegypti *(Fig. 1B). The network includes 4,214 mosquito proteins with 10,209 interactions, of which 62%, 25%, 11% and 2% were inferred from yeast, fly, worm, and more than one reference organism, respectively (Fig. 1B). Among all the proteins in our network, 3,500 proteins are connected into a single interconnected network by 9,719 interactions. The number of interacting partners per node in the interconnected network is 5.55, and the degree distribution fits a power law distribution with a degree exponent of 1.79. As expected, the network also has a high clustering coefficient (0.32) and a short path length (6.46).

### Prediction of mosquito protein function

Cellular functions are often carried out by a group of physically or functionally linked molecules that work together to complete distinct tasks. The high clustering coefficient observed in our network indicates that isolated functional modules may be identified from the network. The Markov Cluster algorithm (MCL) identifies highly connected modules based on simulation of stochastic flow in graphs. It has been shown to be the most robust algorithm for the extraction of protein complexes from protein-protein interaction networks [33]. Using this algorithm, we parsed the mosquito protein interaction network into 494 densely connected clusters (Additional file 3).

**Figure 2:**
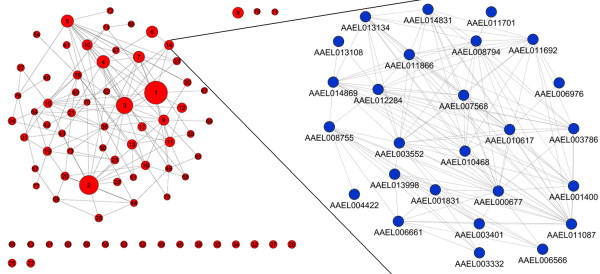
**Functional annotation of 78 clusters comprising more than 10 protein members**. Clusters were identified by a Markov clustering algorithm. Each node represents a cluster, and nodes are connected if there are more than two protein-protein interactions between two clusters. Inset shows a close up view of interactions between component proteins in the cluster 14. Protein members in each of clusters and their enriched GO terms are shown in Additional file 3 and 4, respectively.

We searched for GO biological processes overrepresented in clusters with more than 10 members and functionally annotated 78 clusters (Fig. 2 and Additional file 4). The results indicate that 76% of clusters have one or more enriched GO terms (Benjamini-corrected p value < 0.05). The composition of each cluster is a valuable resource for the annotation of hypothetical proteins in *Ae. aegypti*. For example, cluster 14 comprising 26 proteins represents the RNA polymerase complex (inset of Fig. 2). There are eight hypothetical proteins in this cluster while the remaining eighteen proteins are related to transcription with sixteen of them (61.5%) annotated as DNA-directed RNA polymerase. It is most likely that the eight hypothetical proteins also belong to this functional group. Thus, putative function of hypothetical proteins might be inferred by their proximity to functionally annotated proteins in a cluster. Considering that a large number of *Ae. aegypti *genes are defined as hypothetical proteins, our clustered interaction network provides an useful resource for further annotation of those genes.

### Emerging hypotheses of biological pathways in mosquitoes

Detailed examination of local interaction networks may reveal interconnections of biological pathways and provide further insights on how a biological process is controlled in mosquitoes. Here we focus on the mosquito biological processes that have attracted increasing interest in recent years.

**Figure 3:**
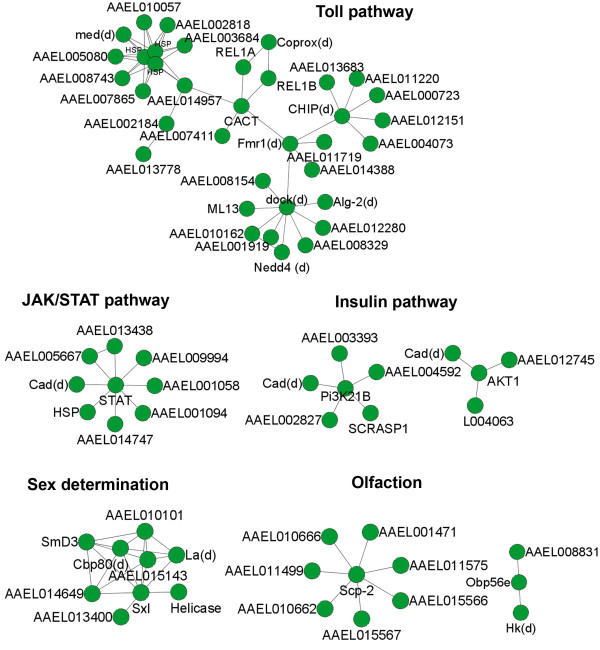
**Local views of mosquito pathways or biological processes**. Genes related to Toll signaling pathway, JAK/STAT pathway, Insulin pathway, sex determination pathway and mosquito olfaction are selected to identify their interaction partners in the network.

The mosquito Toll pathway is involved in defense against fungal and viral infection [2,34]. Activation of the Toll pathway leads to a cascade of events that result in the degradation of CACT, translocation of REL1 transcription factors to the nucleus, and a rapid increase in synthesis of antimicrobial compounds and other effectors [35-37]. As expected, we observed that CACT interacts with both REL1A [VectorBase: AAEL007696] and REL1B [VectorBase: AAEL006930] in the network (Fig. 3). CACT also interacts with AAEL009326, an ortholog of *Drosophila *Fmr1, along with the hypothetical protein AAEL014957. Interestingly, Fmr1 has been shown to be part of the RNAi-related machinery [38]. In *Drosophila*, Fmr1 forms a complex comprising of Argonaute 2 (AGO2) and a *Drosophila *homolog of p68 RNA helicase (Dmp68), both of which are required for efficient RNAi [38]. Considering that both the RNAi and Toll pathways can control dengue infection in mosquitoes, it will be worthwhile to test whether Fmr1 serves as a potential link between the two pathways. Moreover, our network indicates that Fmr1 interacts with dock [VectorBase: AAEL013539], an insulin pathway related gene, which further interacts with an apoptosis-related protein AAEL005910 (*Drosophila *ortholog of Alg-2), an ubiquitin-protein ligase AAEL002536 (nedd-4) and MD2-like receptor AAEL006854 (ML13). In addition, CACT indirectly interacts with three heat shock proteins (Hsp), including AAEL0011704, AAEL0011708, AAEL014843, and one Hsp70-interacting protein CHIP [VectorBase: AAEL012588] (Fig. 3).

STAT is a key component in the JAK/STAT pathway. In the mosquito protein interaction network, it interacts with eight proteins including AAEL014557, an ortholog of *Drosophila *caudal (Cad). Cad also interacts with two proteins associated with the Insulin signaling pathway: Pi3K21B [VectorBase: AAEL013596] and AKT1 [VectorBase: AAEL008823] (Fig. 3). This implies a potential link between the JAK/STAT and Insulin pathways. Further examination of the network found Cad interacting with 49 proteins. Thus, as a hub protein in the networks, Cad may have diverse functions and be essential for the survival of *Ae. aegypti*.

Sex lethal (Sxl) is involved in sex determination in *Drosophila*. In the network, eight proteins interact with *Ae. aegypti *Sxl [VectorBase: AAEL011150]. Two of them are hypothetical proteins. Although no evidence suggests these eight proteins are involved in sex determination, six of them are involved in either RNA binding or RNA helicase activity (Fig. 3). Thus, similar to its *Drosophila *ortholog, Sxl might function as a splicing and translational regulator in mosquito.

Mosquito odorant-binding proteins (Obp) were input into the network to identify their interaction partners. Five odorant-binding proteins and two hypothetical proteins interact with the sterol carrier protein-2 [VectorBase: AAEL002687], a protein which is thought to be involved in intracellular cholesterol transport. OBP56e [VectorBase: AAEL006650] interacts with Hyperkinetic, a gene involved in potassium ion transport, and one hypothetical protein (Fig. 3).

### Prediction and analysis of mosquito responses to dengue infection

A comprehensive mapping of mosquito protein interactions targeted by DENV could provide specific hypotheses and a broad perspective on DENV strategies for replication and persistence in mosquitoes. Previous microarray assays revealed broad responses to DENV infection in mosquitoes that entailed a variety of physiological systems [2]. A recent genome wide RNAi screen in human demonstrated a complex dependence of flaviviruses on host cell physiology, requiring a wide variety of molecules and cellular pathways for successful infection [20]. In addition, studies of physical interactions between flaviviruses and their hosts also provided insight on how viruses hijack the host pathways for their entry and replication. With the mosquito protein interaction network constructed, we can now put all these together and perform an integrated analysis to identify the spectrum of mosquito pathways activated by DENV and other flaviviruses.

#### Prediction of dengue-associated Ae. aegypti proteins

We inferred *Ae. aegypti *proteins associated with DENV infection using three separate datasets. The first dataset was based on RNAi screens in human and *Drosophila *cell lines. Among 82 DENV host factors identified in *Drosophila *with recognizable human homologues, 42 are also human DENV host factors [7]. In addition, 305 human genes have been shown to be associated with WNV, of which 124 also affect DENV infection [20]. Given these virus-associated host genes, we inferred 243 *Ae. aegypti *orthologs which may be involved in DENV infection (Additional file 5). These included 46, 168 and 29 *Ae.aegypti *orthologs from DENV host factors in *Drosophila*, human and both, respectively. Secondly, we predicted 22 mosquito proteins that may directly bind to flavivirus proteins or RNAs based on known host-flavivirus physical interactions collected by literature search (Additional file 6). The third dataset was derived by genome-wide transcriptional profiling of *Ae. aegypti *response to dengue infection. A large number of genes (*n = 456*) in mosquito midgut and the remaining carcass are differentially expressed in response to virus infection [2]. Taken together, we have identified 714 putative dengue associated-mosquito proteins.

There is an overlap between the three datasets with 7 proteins supported by two data resources (Table 1). The latter includes two putative immune related genes: CTLMA12 [VectorBase: AAEL011455] and RM62A [VectorBase: AAEL001317]. CTLMA12 is a member of the gene family C-Type Lectins (CTLs), which are host pathogen-recognition receptors that are specialized in sensing invading pathogens [39]. RM62A is involved in RNAi process http://cegg.unige.ch/Insecta/immunodb, which has been shown to an important pathway through which mosquito defend against dengue infection.

**Table 1:** Putative DENV-associated *Ae. aegypti *proteins supported by more than one experimental evidences

Gene ID	Gene name or description	Physical Interaction	Microarray	RNAi
AAEL001317	RM62A	+	-	+ (WNV)
AAEL002851	tubulin beta chain	+	-	+ (WNV)
AAEL001405	clathrin coat assembly protein	N/A	+	+ (WNV&DENV)
AAEL009360	serine/threonine protein kinase	N/A	+	+ (WNV)
AAEL014368	sap18	N/A	+	+ (WNV)
AAEL011455	CTLMA12	N/A	+	+ (WNV&DENV)
AAEL009565	conserved hypothetical protein	N/A	+	+

The results are derived from RNAi screen, physical interaction assay and microarray assay. Except indicated in parenthesis, data are from DENV-host interactions. +, interactions supported by experiments; -, interaction not supported by experiments; N/A, data are unavailable; DENV, dengue virus; WNV, West Nile virus.

We identified enriched GO terms from the three datasets based on a hypergeometric test with Benjamini and Hochberg multiple testing correction (Table 2). Strikingly, we observed that the same biological processes were overrepresented in RNAi and physical interaction-inferred datasets (Table 2). Two GO terms, protein transport and establishment of localization, were significantly enriched in putative mosquito proteins that bind to viral proteins/RNAs and affect the viral infection (p < 0.05). To initiate infection, DENV binds to and enters host cells via receptor-mediated endocytosis [40]. Physical interaction between the dengue E protein and the mosquito tubulin protein [VectorBase: AAEL002851] has been shown to facilitate internalization of DENV [41], and the RNAi screen provided additional evidence for the role of tubulin in the assembly/transport of the virus particles. DENV also utilizes host proteins for the replication of its genome. The minus-strand 3'UTR of DENV might form the replication complex with calreticulin [VectorBase: AAEL001005], which may actively participate in the process of RNA-dependent RNA synthesis [42]. While RNAi screens investigate the role of host genes in viral infections, microarray profiling shows the expression change of host genes in response to viral infections. Cell death was enriched in the microarray dataset, which was consistent with previous studies indicating that DENV infection induced the expression of mosquito genes involved in apoptosis [2] and resulted in mosquito cell death [43].Our results suggest that different pathways may be involved in the life cycle of viruses and host responses to viral infections.

**Table 2:** Enriched Gene Ontology (GO) biological process terms in three datasets of DENV-associated *Ae. aegypti *proteins

GO Terms	RNAi*	Physical Interaction*	Microarray*
Establishment of Localization	5.0E-4	1.3E-2	NS
Protein Transport	2.4E-2	1.3E-2	NS
Response to stimulus	NS	NS	4.4E-3
Cell death	NS	NS	5.7E-2

Only P values that reach the significant level are shown. * NS, non-significant.

#### Network analysis of putative dengue-associated mosquito proteins

**Figure 4:**
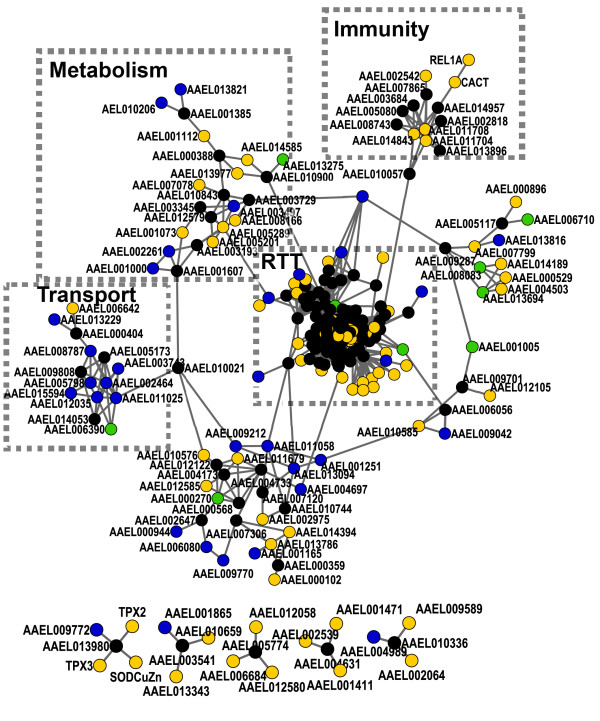
**Putative DENV-associated *Ae. aegypti *protein interaction sub-networks**. One major and five minor sub-networks include virus-associated mosquito proteins inferred from RNAi screen (blue), physical interaction assay (green) and microarray study (yellow), and proteins that connect to at least three of the above proteins (black). Functional analysis of these proteins in the major sub-network highlighted four regions consisting of highly interconnected proteins with closely related functions in each of replication/transcription/translation (RTT), immunity, transport and metabolism. Other regions are comprised of proteins with diverse or unknown functions. Proteins included in the RTT region are listed in Additional file 7.

Among the 714 putative DENV-associated proteins, 280 proteins could be found in the weighted *Ae. aegypti *protein interaction network. We created sub-networks associated with DENV infections by including neighbor proteins that directly connect to at least three of the above DENV-associated proteins. After removing isolated proteins and clusters with less than four members, we identified one major and five minor sub-networks, which contain 121 DENV-associated proteins and 129 neighbor proteins connected by 1,842 interactions (Fig. 4). Further functional analysis of the major sub-network highlighted four regions consisting of highly interconnected proteins with closely related functions in each of replication/transcription/translation (RTT), metabolism, immunity and transport (Fig. 4). To confirm that the above functional groups are more interconnected than the rest of the network, we calculated their graph density in comparison with the density of the global network and random protein groups of the same size taken from the same network. The graph density is defined as the ratio of the number of edges and the number of possible edges in the graph. There are 229 proteins connected by 1,826 edges in the major dengue-associated sub-network with a graph density of 0.07. Functional group RTT consists of 133 proteins connected by 1,655 edges with a graph density of 0.19. Then, we randomly retrieved 133 proteins from the major dengue-associated sub-network and calculated its graph density. Out of 1,000 repeated random sampling, there were only 6 random groups that have graph density more than that of the RTT group, indicating a permutation-based p-value of less than 0.01. Similarly, the graph densities for metabolism, immunity and transport groups were calculated to be 0.14, 0.30 and 0.34, respectively. Their permutation-based p-values are all less than 0.01, supporting the existence of distinct functional groups in the dengue-associated protein interaction network.

RTT is the most complicated region in the major DENV-associated sub-network, and is comprised of 133 proteins (Fig. 4, Additional file 7). Except for 31 proteins with unknown or diverse functions, the largest proportion (92 out of 133; 69.2%) of the proteins in this region plays roles in replication, transcription and translation. This can be explained by DENV hijacking cellular proteins to aid in their own replication and transcription and their reliance on host machinery for their translation. As an example, La protein [VectorBase: AAEL003664] was reported to bind to the 3'and 5' end UTR of viral RNA [23]. La protein has also been shown to inhibit viral RNA synthesis in a dose-dependent manner in mosquito *Ae. albopictus *[24]. As a RNA-binding protein, La is normally located in the nucleus. It has to be redistributed to the cytoplasm of dengue-infected cells to play its role in the viral replication process. It is still unknown how to trigger the export of La protein from the nucleus to the cytoplasm for the binding with the 3'and 5' UTR and regulation of viral RNA synthesis.

Metabolism is another function that was highlighted in one region of the sub-network comprising highly interconnected proteins (Fig. 4). 15 out of 23 proteins (65.2%) in this region function in metabolism. They include genes that express enzymes to catalyze the reactions in the biosynthesis pathway of L-arginine (AAEL003345), aspartate, phenylalanine (AAEL012579), isoprenoid (AAEL003497) and tetrahydrofolate (AAEL002261). Other proteins in this region are involved in metabolic process of galactose (AAEL001607), phosphorus (AAEL003193), malate (AAEL008166, AAEL001073), lyxose (AAEL013821, AAEL010206), mevalonate pathway I (AAEL005201) and arginase pathway (AAEL005289). As a parasite, DENV depends on the metabolic network of the host cell to provide the energy and macromolecule subunits necessary for their replication. Consequently, host infected by the virus can produce dramatic metabolic alterations. Interestingly, three proteins (AAEL012579, AAEL003497 and AAEL005201) that are related to lipid metabolism were found in this group. Further studies are needed to study the impact of DENV on mosquito metabolism, especially lipid metabolism, and the effects of host metabolic perturbation on DENV replication in mosquitoes.

Immunity is the third function that was highlighted in the major DENV-associated sub-network. A region with highly interconnected proteins consists of the Toll pathway genes and heat shock proteins (Fig. 4). It includes REL1, CACT and three heat shock proteins [VectorBase: AAEL011704, AAEL011708 and AAEL014843]. Highlighting of this Toll pathway-related proteins is consistent with our previous report that the *Ae. aegypti *Toll pathway can control dengue infection [2]. Previous studies also show that the Onyong-nyong virus induces expression of the heat shock protein cognate 70B in *An. gambiae*, which has an inhibitory effect on the replication of this virus [44]. It will be interesting to explore whether the Toll pathway can regulate the expression of heat shock proteins. There are eight proteins that have never been reported to be associated with dengue infection in this region. Three of them are hypothetical proteins. The others are AAEL008743, AAEL005080, AAEL013896, AAEL002818 and AAEL003684. Among them, AAEL002818 may be related to immune defense. Knock-down of its *Drosophila *ortholog, U2af50, can induce the expression of immune genes [45].

Fourteen highly interconnected proteins are included in the region with a main function in transport (Fig. 4). Ten of them are involved in ion transport while additional four encode tubulin alpha or beta chains, which can mediate microtubule-based movement. Previous studies found that Sindbis viruses induce dramatic changes in the expression of mosquito genes involved in ion transport processes [46].

**Figure 5:**
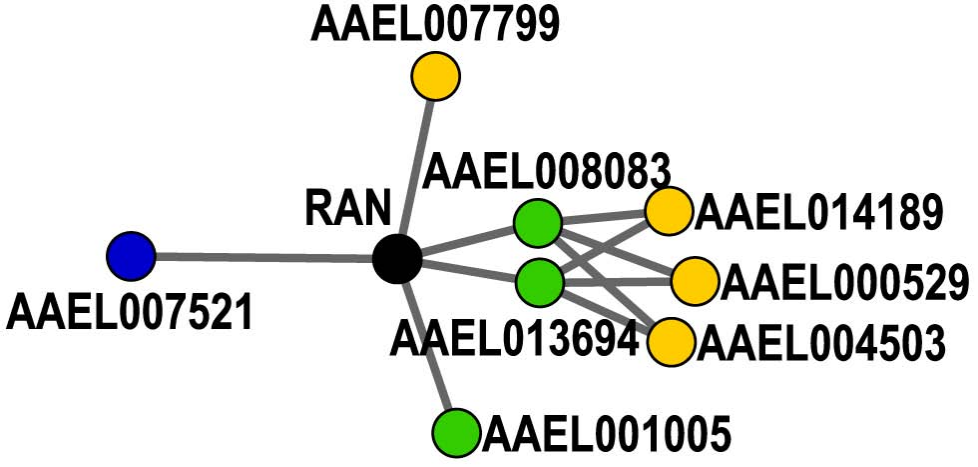
**One neighbor protein interacts with a high number of DENV-associated proteins derived from three datasets**. Interactions of Ras-related nuclear protein (RAN) with five DENV-associated proteins highlight its role in viral infections. The node color scheme is same as used in the Fig. 4.

In the DENV-associated sub-network, some neighbor proteins interact with a high number of DENV-associated proteins derived from the above three datasets. These proteins are highly like to involve in DENV-mosquito interactions. For example, AAEL009287 is a Ras-related nuclear protein (RAN) involved in the transport of large molecules across the nuclear envelope. In the sub-network, RAN is connected to five putative DENV-associated proteins (Fig. 5). Ribosomal protein p40 [VectorBase: AAEL008083/AAEL013694] is a precursor of the high affinity laminin receptor, a putative receptor for the entry of DENV into host cells [25,47]. While mature laminin receptors are located on the cell surface, p40 has been detected in different compartments of cells, and is suggested to be a shuttle protein between the cytoplasm and the nucleus [48]. Calreticulin [VectorBase: AAEL001005] may bind to the 3'UTR of DENV RNA [42]. It has to be retrotranslocated from endoplasmic reticulum lumen to cytosol for this gene regulatory function [49]. RAN exists in the cell in two nucleotide-bound forms, RanGDP and RanGTP, whose concentration is controlled by the regulator of chromosome condensation (RCC) [VectorBase: AAEL007799]. RAN interacts with importin [VectorBase: AAEL007521] and exportin to change their ability to bind or release cargo molecules for the transport through the nuclear pore. The interactions of RAN with these mosquito proteins suggest that RAN may work together with RCC, importin, and p40 to redirect viral proteins to the nucleus, and then help the translocation of calreticulin, La, and other RNA-binding proteins to the cytoplasm to facilitate viral genome replication. This hypothesis is supported by the previous studies indicating that RAN was targeted by vesicular stomatitis virus to block transport of RNAs and proteins between the nucleus and cytoplasm of *Xenopus laevis *oocytes [50]. Further studies are required to elucidate the roles that mosquito RAN protein play in the life cycle of DENV.

### Validation of the predicted dengue-mosquito interactions in *Ae. aegypti*

**Figure 6:**
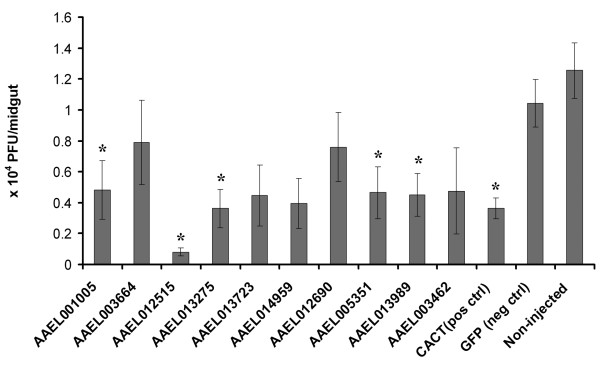
**Validation of dengue-mosquito interactions in *Ae. aegypti *for the selected genes by RNAi**. Dengue virus loads were assayed in the midguts of gene silenced mosquitoes, GFP dsRNA treated and non-injected control mosquitoes. The infection was measured by plaque assay in C6/36 cell and the value was shown as viral titer (PFU) per midgut. *, P < 0.05, in Mann-Whiteney U test compared to GFP control. Primary data for the plaque assay are presented in Additional file 8. Pos ctrl, positive control; neg ctrl, negative control.

To verify the predicted dengue-mosquito interactions, we randomly selected ten mosquito genes from the above DENV-associated mosquito proteins and tested their influences on dengue infection in *Ae. aegypti *using RNAi knockdown. Three days before mosquitoes were fed with dengue-infected blood, double stranded RNA (dsRNA) of each of these genes was injected into the thorax of mosquitoes. dsRNA of CACT and GFP were injected as positive and negative controls, respectively. Non-injected mosquitoes were used as an additional negative control. Seven days post-infection, mosquito midguts were dissected and the viral titers were measured by plaque assay. As a result, silencing of five out of ten genes (50.0%) led to a significant reduction of DENV infection in the midgut compared to the GFP dsRNA control (Mann-Whiteney U test, P < 0.05). The anti-dengue effect was also observed in the Cactus dsRNA treatment. There is no significant difference in dengue titer between non-injected and GFP dsRNA injected mosquitoes (Fig. 6 and Additional file 8). These above results support the reliability of our predictions.

Notably, knockdown of a putative tumor suppressor protein [VectorBase: AAEL012515] led to a 13-fold decrease in DENV infection in the mosquito midguts (Fig. 6). The *Drosophila *ortholog of AAEL012515, TSG101, is the negative regulator of the JAK/STAT pathway and functions as a ubiquitin-protein ligase [51]. The JAK/STAT pathway has been demonstrated to be required for anti-viral defense in *Drosophila *[52]. In humans, silencing of PIAS2, the protein inhibitor of activated STAT, can also reduce both DENV and WNV infections [20]. The above results strongly suggest that the JAK/STAT pathway plays an important role in defense against dengue infection in mosquitoes. Our previous studies also show that several important components of this pathway, including DOME [VectorBase: AAEL012471], were up-regulated in response to dengue infection in *Ae. aegypti *[2]. Thus, the JAK/STAT pathway might play a role in regulation of dengue infection in mosquitoes.

Silencing of the other four genes, AAEL001005, AAEL013989, AAEL005351 and AAEL013275, also significantly reduced the dengue infection in mosquito midguts (Fig. 6). AAEL001005 is a putative mosquito calreticulin protein, whose ortholog in human has been shown to form a complex with 3'UTR in the minus-strand RNA of DENV [42]. Considering that the regulatory sequences involved in viral replication reside in both ends of the flavivirus genome, it is very likely that calreticulin plays a role in the replication of DENV. AAEL013989 is a putative protein translocation complex beta subunit with protein transporter activity. This gene was induced in *Ae. aegypti *in response to Sindbis virus infection [46]. Silencing of its *Drosophila *and human ortholog, Sec61β, significantly reduced the dengue infection in S2 cell line and HuH-7 cells, respectively [7]. AAEL005351 is Leucine-rich transmembrane protein. Similarly, its *Drosophila *and human orthologs were proven to be required for dengue infection [7]. Importin beta-1, AAEL013275, is also a protein with transmembrane transporter activity. Previous studies found that dengue virus NS5 is able to interact with the nuclear import receptor importin-β although the roles of this interaction is still unclear.

Our RNAi results indicate the notable conservation of required factors between mosquito and human hosts. As described in the above, all the five genes verified here are known to play important roles in interactions of DENV with human host. It appears that common host factors present in human and mosquito, and their interactions with DENV may determine the high efficiency of DENV in entry and replication within these two distantly related animals. In order to maintain the transmission cycle of DENV between mosquito and human, a perfect match in the cellular components and conserved DENV-host interactions may be required between the two host species. Our study makes the first steps towards identification of common pathways that are hijacked by DENV in humans and mosquitoes. A recent study using a genome-wide RNA interference screen in *D. melanogaster *cells found that 51.2% of the human homolog of dengue associated insect host factors were also required for dengue infection in human [7]. This is consistent with our results that 50.0% mosquito orthologs of dengue human host factors are required for its infection in mosquito.

Our studies also show that the protein interaction network is a powerful tool in the study of vector biology. It can not only provide an additional tool for further annotation of vector genome, but can also serve as a novel platform to integrate datasets from different experiments in order to dissect complicated biological processes in insect vectors. By integrating the results from high-throughput assays such as genome-wide RNAi screens, microarrays and yeast two-hybrid system, a protein interaction network can significantly increase prediction accuracy. Moreover, multiple and concurrent signal-transduction pathways operate and are deregulated during the course of vector-pathogen interactions. Such highly interconnected and temporally and spatially regulated signaling pathways can only be dissected by system-level approaches. Construction of a protein interaction network can lay a foundation to study the system biology of pathogen-vector interactions, as shown in our integrated analysis of DENV-associated mosquito proteins and mosquito protein interaction network.

As a first step, we used protein interactions data from model organisms to infer a mosquito protein interaction network. In order to understand the unique biological processes in mosquitoes, it is necessary to construct the network using experimental data directly from mosquitoes, such as a high-throughput yeast two-hybrid system. Such an independent mosquito network will allow us to compare and contrast the network's response to dengue infection between humans and mosquitoes. This can reveal unique features of dengue-host interactions in these two species, which will help us understand why DENV causes serious disease in human but no deleterious effects in the mosquito vector.

Arboviruses pass through two different hosts in their life cycle, with great benefit taken from vectors for their mobility, diffusion and evolution. From an implementation standpoint, this also provides unique opportunity for identification of weak linkages in their life history to target using antiviral agents. For example, conserved DENV host factors may be used as targets for anti-dengue agents for blocking dengue transmission in mosquitoes or for pharmacological intervention in humans. With the genome available, insect vectors may be used as an animal model to study the basic biology of arboviruses, and develop novel therapeutics or control strategies.

## Conclusions

We have developed a mosquito protein interaction network based on large-scale protein interaction datasets in yeast, worm, and fly. The weighted interaction network includes 4,214 mosquito proteins with 10,209 interactions, among which 3,500 proteins are connected into a single interconnected network by 9,719 interactions. This network not only provides a valuable resource for the further annotation of the *Ae. aegypti *genome, but also makes the first step towards studies of mosquito systems biology. In this regard, we identified a cluster of Toll pathway related protein interactions, and predicted the potential link between the Toll pathway, RNAi pathway and expression of heat shock proteins. We then used datasets from physical interaction assays, RNAi and microarray to infer *Ae. aegypti *proteins associated with dengue infection. This led to the identification of 714 putative dengue-associated mosquito proteins. An integrated analysis of these proteins in the network highlighted four regions consisting of highly interconnected proteins with closely related functions in each of replication/transcription/translation (RTT), immunity, transport and metabolism. Finally, by knocking down the predicted DENV-associated mosquito proteins with RNAi, we successfully verified that five of ten candidate genes were required for DENV infection. Our results support the presence of common host requirement of DENV in human and mosquito.

## Methods

### Prediction of protein-protein interactions in *Ae. aegypti*

We retrieved three high-throughput protein interaction datasets in model organisms along with their confidence scores [12,13,29]. InParanoid was used to determine homology between *Ae. aegypti *and three model organisms, *S. cerevisiae, D. melanogaster*, and *C. elegans *[30]. Proteins A and B in *Ae. aegypti *are predicted to interact if their respective orthologs A' and B' are interacting partners in a model organism. We use IP(AA') and IP(BB') to denote InParanoid scores for ortholog pairs AA' and BB', CS(A'B') to indicate confidence values of protein interactions in the model organism. Then, the confidence of predicted interactions in *Ae. aegypti*, CS(AB) is CS(A'B') × average of IP(AA') and IP(BB'). If orthologs of proteins A and B in more than one model organism show evidence of interaction, we will have more than one confidence score for A and B. In this case, the maximum value is taken as interaction confidence.

We then retrieved GO terms for all *Ae. aegypti *proteins based on the TIGR annotation pipeline [53]. GO is comprised of three subcategories, biological process (BP), molecular function (MF), and cellular component (CC). Semantic similarity values were calculated for each predicted interacting protein pair in terms of BP, MF and CC using the *SemSim *package in BioConductor http://www.bioconductor.org/packages/2.2/bioc/vignettes/SemSim/inst/doc/SemSim.pdf. Specifically, Resnik's approach is applied to calculate GO term similarity based on the assumption that the more information two terms share, the more similar they are [54]. The shared information of two terms is indicated by the information content of their parents, defined as the frequency of each term, or any of its children, occurring in an annotated dataset. Less frequently occurring terms are said to be more informative. GO term similarity defines semantic similarity of proteins annotated with these terms. In the case of multiple annotations for a protein, maximum term similarity is taken as the protein similarity [32].

### Network analysis of weighted protein interactions

Global measures of network topology provide quantitative insight into biological systems. We used the igraph package in the R computing environment to analyze the degree distribution, clustering coefficient, characteristic path length, and diameter of our network. Then, Markov Clustering algorithm (MCL) was applied to partition our mosquito interaction network into densely connected clusters [55], which were displayed and analyzed using the GenePro plugin in Cytoscape [56]. GenePro displays protein clusters as individual nodes interconnected through interacting proteins. Each individual cluster may be expanded to allow detailed analysis in terms of component proteins and their interactions in the context of the full network.

### Prediction and analysis of dengue-*Ae. aegypti *mosquito protein interactions

Known physical interactions between flavivirus proteins/RNA and their host proteins were collected by literature search. If an ortholog can be identified in *Ae. aegypti *for the host protein, then the mosquito protein and corresponding virus protein/RNA are predicted to interact. Both *Inparanoid *and Ensembl were used for ortholog identification, and only the mosquito protein(s) with the highest *Inparanoid *score were used for downstream analysis. Similarly, mosquito orthologs were identified for a set of human and *Drosophila *genes associated with DENV and WNV that were derived from RNA interference screen [20]. Then, a hypergeometric test of GO annotations was used to discover enriched functions in the predicted sets of mosquito proteins using the BinGO plugin [57] in Cytoscape. These proteins were also mapped to the mosquito protein interaction network and the resulting subnetwork was visualized by Cytoscape. The functional classification of mosquito proteins in each module was performed as described previously [53]. The graph density of each functional group or random protein groups with the same size was calculated by graph.density function in an R package igraph http://igraph.sourceforge.net/doc/R/graph.density.html.

### Mosquito rearing and cell culture maintenance

*Ae. aegypti *mosquitoes of the Rockefeller/UGAL strain were maintained on a sugar solution at 27°C and 85% humidity with a 12-hr light/dark cycle according to standard rearing procedures. The *Ae. albopictus *cell line C6/36 was grown in minimal essential medium (MEM) with 10% heat inactivated FBS, 1% L-glutamine, and 1% non-essential amino acids at 32°C with 5% CO_2_.

### Gene-silencing assays

DsRNA were synthesized from PCR-amplified gene fragments using the T7 Megascript kit (Ambion). The sequences of the primers are listed in Additional file 9. RNAi-based gene-silencing assays were conducted as previously reported: Approximately 69 nl dsRNAs (4 mg/ml) in water were injected into the thorax of cold-anesthetized 4-day-old female mosquitoes using a nano-injector [2]. Three to four days after injection, mosquitoes were fed on a DENV-2-supplemented blood meal.

### DENV-2 infections

The New Guinea C strain of DENV-2 was propagated in C6/36 cells according to standard conditions. In brief, 0.5 ml aliquots of virus stock were used to infect 75-cm2 flasks of C6/36 cells at 80% confluency with a multiplicity of infection (MOI) of 3.5 virus particles/cell. Infected cells were incubated for 5-7 days. Cells were harvested with a cell scraper and lysed to release the virus particles by repeated freezing and thawing in dry CO_2 _and a 37°C water bath. The virus suspension was mixed 1:1 with commercial sheep blood. The blood meal (with 2 × 10^7 ^PFU/ml DENV) was maintained at 37°C for 30 min prior to feeding 6- to 7-day-old mosquitoes http://www.jove.com/index/Details.stp?ID=220. We confirmed that the virus titer we used resulted in a 90% infection rate in experimentally infected females by an indirect fluorescent antibody assay performed on head squashes at 14 days after the infectious meal.

### Mosquito dissection and Plaque assay

Seven days after blood meal, mosquitoes were briefly washed in 70% ethanol, then rinsed in sterile distilled water. The midguts were dissected in sterile PBS and transferred separately to microcentrifuge tubes containing 150 ul of MEM, then homogenized with a Kontes pellet pestle motor in a sterile environment. Each replicate contain 4-5 midguts. At least three independent biological replicate assays were performed for each gene.

### Plaque assay

The virus-containing homogenates were serially diluted and inoculated into C6/36 cells in 24-well plates. After incubation for 5 days at 32°C and 5% CO2, the plates were assayed for plaque formation by peroxidase immunostaining, using mouse hyperimmune ascitic fluid (MHIAF, specific for DENV-2) and a goat anti-mouse HRP conjugate as the primary and secondary antibody, respectively.

## List of abbreviations

DENV: dengue viruses; JVE: Japanese encephalitis virus; WNV: West Nile; CACT: Cactus; UTR: untranslated regions; HCV: Hepatitis C virus; RNAi: RNA interference; GO: Gene Ontology; BIND: Biomolecular Interaction Network Database; RTT: replication/transcription/translation; CTL: C-Type Lectins.

## Authors' contributions

Conceived and designed the project: XG, ZX. Analyzed the data: XG, ZX and YXI. Performed the experiments: YXU, GB and AP. Contributed reagents/materials/analysis tools: XG, BG, YXI. Wrote the paper: XG, ZX. All authors read and approved the final manuscript.

## Supplementary Material

Additional file 1 **Predicted protein-protein interactions in *Ae. aegypti***. Click here for file

Additional file 2 **Semantic similarity and interaction confidence of putative interacting proteins predicted from yeast alone**. Protein interactions were binned based on their confidence scores. The average semantic similarity values in each bin were shown for all three GO sub-categories. Percentage of interactions in each bin was shown as a histogram bar. Click here for file

Additional file 3 **Clusters in the high-confidence protein interaction network identified by Markov Clustering Algorithm**. Click here for file

Additional file 4 **Functional annotation of 78 clusters comprising more than 10 protein members**. Click here for file

Additional file 5 **Putative DENV-associated *Ae. aegypti *proteins based on RNAi screen results**. Click here for file

Additional file 6 **Putative DENV-associated *Ae. aegypti *proteins based on physical interaction data**. Click here for file

Additional file 7 **List of highly interconnected proteins in one region of sub-networks with a main function in replication/transcription/translation (RTT)**. Click here for file

Additional file 8 **Averaged data from three independent biological replicate plaque assays of the virus titer in the midguts of the gene silenced and GFP dsRNA treated mosquitoes**. S.E., standard error; S, significant; NS, Non-significant. Click here for file

Additional file 9 **The prime sequences used for amplification of the target genes**. T7 promoter sequence (TAATACGACTCACTATAGGG) is added at the 5' end of all the primers for synthesis of the dsRNAs. Click here for file
