# Supplementary figures and images for: Response of the mosquito protein interaction network to dengue infection
Source: BMC Genomics. 2010 Jun 16;11:380. doi: 10.1186/1471-2164-11-380 (PMC3091628; doi:10.1186/1471-2164-11-380)

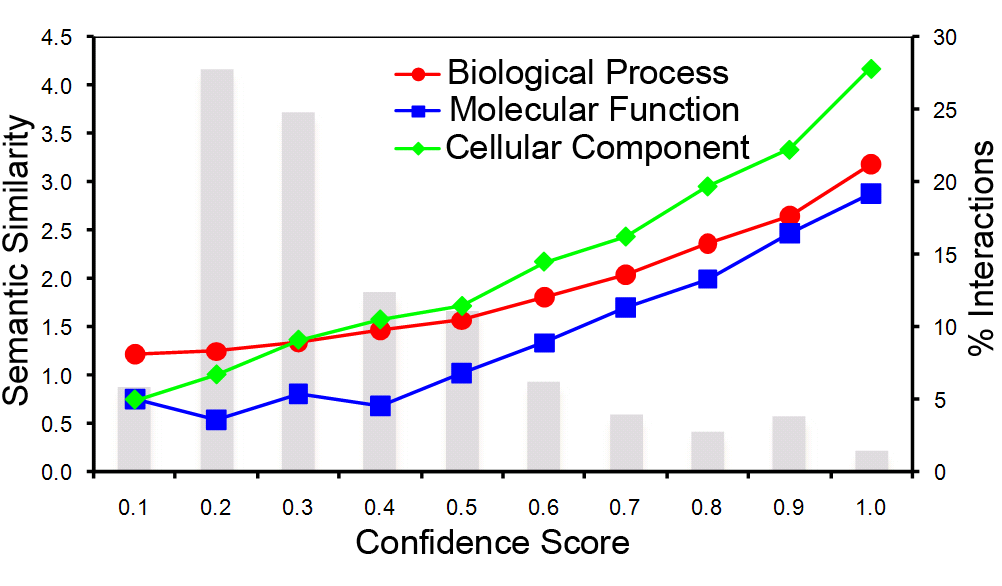

Supplement: Additional file 2 — Semantic similarity and interaction confidence of putative interacting proteins predicted from yeast alone. Protein interactions were binned based on their confidence scores. The average semantic similarity values in each bin were shown for all three GO sub-categories. Percentage of interactions in each bin was shown as a histogram bar. [file 1471-2164-11-380-S2.TIFF]
