# Supplementary material for: Response of the mosquito protein interaction network to dengue infection
Source: BMC Genomics. 2010 Jun 16;11:380. doi: 10.1186/1471-2164-11-380 (PMC3091628; doi:10.1186/1471-2164-11-380)
Supplement: Additional file 8 — Averaged data from three independent biological replicate plaque assays of the virus titer in the midguts of the gene silenced and GFP dsRNA treated mosquitoes. S.E., standard error; S, significant; NS, Non-significant. [file 1471-2164-11-380-S8.DOC]

**Additional file 8. Averaged data from three independent biological replicate plaque assays of the viral titer in the midguts of the gene silenced and GFP dsRNA treated mosquitoes. S.E., standard error; S, significant; NS, Non-significant**

| **Treatment** | **Midgut #** | **Mean PFU ± S.E., x 10 4/ midgut** | **P value** | **Significance** |
| --- | --- | --- | --- | --- |
| dsGFP | 40 | 1.04 ± 0.15 | ─ | ─ |
| Non-injected | 30 | 1.26 ± 0.18 | 0.33 | NS |
| dsCactus | 30 | 0.36 ± 0.07 | 0.01 | S |
| AAEL001005 | 30 | 0.48 ± 0.19 | 0.04 | S |
| AAEL003664 | 15 | 0.79 ± 0.27 | 0.47 | NS |
| AAEL012515 | 15 | 0.08 ± 0.03 | 0.02 | S |
| AAEL013275 | 30 | 0.36 ± 0.13 | 0.01 | S |
| AAEL013723 | 15 | 0.45 ± 0.20 | 0.08 | NS |
| AAEL014959 | 15 | 0.40 ± 0.16 | 0.05 | NS |
| AAEL012690 | 30 | 0.76 ± 0.22 | 0.27 | NS |
| AAEL005351 | 30 | 0.47 ± 0.17 | 0.03 | S |
| AAEL013989 | 30 | 0.45 ± 0.14 | 0.04 | S |
| AAEL003462 | 30 | 0.48 ± 0.28 | 0.08 | NS |

S, significant; NS, non-significant
