# Supplementary material for: Response of the mosquito protein interaction network to dengue infection
Source: BMC Genomics. 2010 Jun 16;11:380. doi: 10.1186/1471-2164-11-380 (PMC3091628; doi:10.1186/1471-2164-11-380)
Supplement: Additional file 9 — The prime sequences used for amplification of the target genes. T7 promoter sequence (TAATACGACTCACTATAGGG) is added at the 5' end of all the primers for synthesis of the dsRNAs. [file 1471-2164-11-380-S9.DOC]

**Additional file 9. The prime sequences used for amplification of the target genes. T7 promoter sequence (TAATACGACTCACTATAGGG) was added at the 5’ end of all the primers for synthesis of the dsRNAs.**

| **Transcript ID** | **Direction** | **Primer Sequence** |
| --- | --- | --- |
| AAEL013275-RA | Forward | 5’-CGGAGGATATGCGAGAATACG-3’ |
|  | Reverse | 5’-TATGCTGGTAGTAGAGGGTGAG-3’ |
| AAEL013723-RA | Forward | 5’-CAACAAGAAGGATTCAGCACTC-3’ |
|  | Reverse | 5’-ACACGTAGATGGTTCGATTCC-3’ |
| AAEL001005-RA | Forward | 5’-GCCAAGTTCACACCCTTCTC-3’ |
|  | Reverse | 5’-CATTCGCCGTCCATCTCATC-3’ |
| AAEL014959-RA | Forward | 5’-ACAGAGCACTGACCGTATCG-3’ |
|  | Reverse | 5’-TGCCCATATTGCCCATATTTCC-3’ |
| AAEL012515-RA | Forward | 5’-TCAACGACGGAACGGTGAAG-3’ |
|  | Reverse | 5’-TGGCATGTACGGTGGATAGC-3’ |
| AAEL001382-RA | Forward | 5’-ACTCAGCCACGAAACGGTTAC-3’ |
|  | Reverse | 5’-CCTTGGTCCACCACGTCCTC-3’ |
| AAEL003664-RA | Forward | 5’-TCCAGAGCAGAACGAAGAGAC-3’ |
|  | Reverse | 5’-GTCAATGAAGGCAACCTCCAG-3’ |
| AAEL000709-RA | Forward | 5’-CGAGTCAACAGAACCCGAGCAG-3’ |
|  | Reverse | 5’-TGGCCCGTCAGCACCGAAAG-3’ |
| AAEL003462-RA | Forward | 5’-CTCGGGCGCGGGCTATCAAG-3’ |
|  | Reverse | 5’-GCGGACGTTGTTCCGACCGT-3’ |
| AAEL005351-RA | Forward | 5’-GAGCCGCCGGCAAGTACCAG-3’ |
|  | Reverse | 5’-ACACCAGCCGCATTCTCGGC-3’ |
| AAEL012690-RA | Forward | 5’-GGCCGTACGGGATGGCGAAG-3’ |
|  | Reverse | 5’-CGCTGCTGCTGGTGAACCGA-3’ |
| AAEL013989-RA | Forward | 5’-ACGTGGATTCCCAAAGTGGCGT-3’ |
|  | Reverse | 5’-GGGCAGCAGTCGTCGTGGTC-3’ |
